# Impact of methoxyacetic acid on mouse Leydig cell gene expression

**DOI:** 10.1186/1477-7827-8-65

**Published:** 2010-06-18

**Authors:** Gargi Bagchi, Yijing Zhang, David J Waxman

**Affiliations:** 1 Division of Cell and Molecular Biology, Department of Biology, Boston University, Boston, MA 02215, USA

## Abstract

**Background:**

Methoxyacetic acid (MAA) is the active metabolite of the widely used industrial chemical ethylene glycol monomethyl ether, which is associated with various developmental and reproductive toxicities, including neural toxicity, blood and immune disorders, limb degeneration and testicular toxicity. Testicular toxicity is caused by degeneration of germ cells in association with changes in gene expression in both germ cells and Sertoli cells of the testis. This study investigates the impact of MAA on gene expression in testicular Leydig cells, which play a critical role in germ cell survival and male reproductive function.

**Methods:**

Cultured mouse TM3 Leydig cells were treated with MAA for 3, 8, and 24 h and changes in gene expression were monitored by genome-wide transcriptional profiling.

**Results:**

A total of 3,912 MAA-responsive genes were identified. Ingenuity Pathway analysis identified reproductive system disease, inflammatory disease and connective tissue disorder as the top biological functions affected by MAA. The MAA-responsive genes were classified into 1,366 early responders, 1,387 mid-responders, and 1,138 late responders, based on the time required for MAA to elicit a response. Analysis of enriched functional clusters for each subgroup identified 106 MAA early response genes involved in transcription regulation, including 32 genes associated with developmental processes. 60 DNA-binding proteins responded to MAA rapidly but transiently, and may contribute to the downstream effects of MAA seen for many mid and late response genes. Genes within the phosphatidylinositol/phospholipase C/calcium signaling pathway, whose activity is required for potentiation of nuclear receptor signaling by MAA, were also enriched in the set of early MAA response genes. In contrast, many of the genes responding to MAA at later time points encode membrane proteins that contribute to cell adhesion and membrane signaling.

**Conclusions:**

These findings on the progressive changes in gene expression induced by MAA in a cultured Leydig cell model may help elucidate signaling pathways that lead to the testicular pathophysiological responses induced by MAA exposure and may identify useful biomarkers of MAA toxicity.

## Background

Methoxyacetic acid (MAA) is the primary active metabolite of the industrial chemical ethylene glycol monomethyl ether, a component of paints, inks, varnishes and anti-icing additive in jet fuels [1]. MAA exposure is associated with various developmental and reproductive toxicities in both rodents and humans, including decreased sperm production reflecting increased apoptosis of primary spermatocytes [2] and is accompanied by gene expression changes in germ cells (reviewed in [3]). However, the precise testicular cell target(s) of MAA that lead to the observed increase in germ cell apoptosis are uncertain. The survival and proper functioning of germ cells requires cooperation of several testicular cell types, including Sertoli cells, which nurture the developing germ cells through spermatogenesis [4], and Leydig cells, the major site of testosterone production in males [5]. MAA-induced changes in gene expression in Sertoli and Leydig cells could therefore have a significant impact on germ cell behavior and overall reproductive function. While MAA-induced changes in Sertoli cell gene expression have been described [6], the impact of MAA on Leydig cell gene expression has not been investigated.

Environmental chemicals that interfere with normal Leydig cell gene expression have the potential to impact germ cell function. Leydig cell lines have been useful for investigating the testicular actions of environmental chemicals, including effects on gene expression [7], and in the case of MAA, changes in gene expression have been investigated using the cultured TM3 Leydig cell model, which is derived from the testis of the immature Balb/c mouse [8]. In particular, MAA was found to alter the expression of TM3 cell genes involved in testosterone biosynthesis (*Cyp17a1*) and androgen binding (*Shbg*) [9]. Presently, we use this same Leydig cell model to characterize the sequential changes in gene expression that occur following MAA exposure. Genome-wide transcriptional profiling was carried out to elucidate the global impact of MAA treatment at each of three time points (3, 8 and 24 h) in an effort to identify both early (primary) and late (secondary) MAA response genes. Cells were treated with MAA at 5 mM, corresponding to the plasma concentration associated with ethylene glycol monomethyl ether-induced germ cell toxicity in mice [10], and at 1 mM, to identify genes affected at a concentration of MAA similar to that seen in exposed humans [11]. A total of 3,912 genes responsive to 5 mM MAA treatment were identified, 1,629 of which were also responsive at 1 mM MAA. The early MAA-responsive genes include 106 genes involved in transcriptional regulation, whereas many of the genes responding to MAA at later time points encode membrane proteins that contribute to cell adhesion and membrane signaling. These MAA-induced perturbations of cellular and biological functions may help elucidate the signaling pathways perturbed by this environmental toxicant and explain its mechanism of action at the gene level.

## Methods

### Chemicals and reagents

MAA and horse serum were purchased from Sigma Chemical Co. (St. Louis, MO). DMEM-F12 culture medium, fetal bovine serum (FBS) and TRIzol reagent were purchased from Invitrogen Corp. (Carlsbad, CA).

### MAA treatment of TM3 cells and RNA analysis

Mouse TM3 Leydig cells (American Type Culture Collection, Manassas, VA) were grown in DMEM-F12 medium containing 5% horse serum and 2.5% FBS. Cells were grown to ~60% confluence and treated with culture medium alone, or with culture medium containing 1 mM or 5 mM MAA for either 3, 8 or 24 h. Total RNA was then isolated using TRIzol reagent, followed by incubation with RQ1 RNAse-free DNAse for 1 h at 37°C and then heat inactivation at 75°C for 5 min. A total of 6 cultures of TM3 cells were independently treated with MAA under each of the 6 treatment conditions specified above (i.e., 1 mM or 5 mM MAA for either 3, 8 or 24 h), and the corresponding 36 RNA samples were validated by RNA integrity analysis (Agilent Bioanalyzer). Each RNA sample was also validated in terms of the MAA response by qPCR analysis using SYBR Green I-based chemistry [12] and primers specific for 3 genes known to respond to MAA (*Cyp17a1*, *Shbg*, and *Igfbp3; *[9]) to verify consistency of the MAA responses. Dissociation curves were examined after each qPCR run to ensure amplification of a single, specific product at the correct melting temperature. The 6 RNA samples for each treatment condition were then used to prepare two independent pools (n = 3 RNA samples each) for microarray analysis with dye swaps, as described below. Microarray results were validated for 6 genes, three of which were induced at both 1 mM and 5 mM MAA, and three of which were repressed at 5 mM MAA. Data are presented as the expression of the gene of interest relative to an 18 S RNA internal control in the MAA-treated sample compared with the untreated control, as determined using the comparative Ct method [13], and are based on duplicate RNA samples for each of three independent experiments for each condition of MAA treatment, with all six samples assayed in triplicate (mean ± SE for n = 3).

### Microarray analysis

The Agilent Whole Genome Mouse Microarray platform (catalog G4122F; Agilent Technology) was used to characterize MAA-induced changes in TM3 cell gene expression. This array contains 41,174 mouse probes (features), each consisting of a 60-mer oligonucleotide. Accession numbers were obtained for 39,355 out of the 41,174 probes, of which 33,011 were assigned gene names. An additional 3,570 probes were assigned gene names using the microarray probe annotation tool AILUN [14] to map microarray probes to Entrez genes [15]. Each probe corresponding to a distinct mouse transcript is referred to as representing a separate gene/gene product. RNA samples pooled from 3 separate TM3 cell cultures were analyzed in a total of six separate competitive hybridization experiments, corresponding to the above specified combinations of exposure time (3 h, 8 h, and 24 h) and MAA concentration (1 mM, 5 mM). Sample labeling, hybridization to microarrays, scanning and calculation of normalized expression ratios were carried out at the Wayne State University Institute of Environmental Health Sciences microarray facility using Alexa 555 and Alexa 647 aminoallyl-aRNA samples as described [16]. Dye swap experiments were carried out using a separate pool of three MAA-treated TM3 cultures. To carry out the dye swaps, Alexa 555-labeled RNA from one of the MAA-treatment conditions was mixed with Alexa 647-labeled RNA from the untreated control at the same time point, and vice versa. TIFF images of each scanned slide were analyzed using Agilent's feature extraction software with linear and LOWESS normalization followed by combination of dye-swap samples and calculation of weighted ratios and *p*-values based on the Rosetta error model using Rosetta Resolver (version 5.1, Rosetta Biosoftware, Seattle, WA) [17]. Features flagged in either channel were excluded from analysis. The final set of expression ratios and *p-*values is available as GEO series GSE-20625.

### Statistical analysis

In those cases where two or more probes mapped to the same gene accession and gave the same pattern of expression across all microarrays (reflecting probe redundancy in the array platform), a single representative probe was retained in the final data set. Of the 41,174 probes included on the array, 33,940 non-redundant probes were identified for the 5 mM MAA treatment data set. The statistical significance of differential expression of each corresponding transcript was determined by application of a filter (*p *< 0.005) to the Rosetta-generated *p-*values. Next, an absolute log_2 _ratio filter of >2 SD above the mean value was combined with the above *p*-value filter to determine the number of gene transcripts that were differentially regulated at any of the three time points. In total, 3,912 probes met the combined thresholds for differential expression in at least one of the three 5 mM MAA time points. Of the 33,940 non-redundant probes, 5,031 met the >2 SD differential expression filter for at least one of the three microarray comparisons. The number of probes expected to meet the combined threshold (*p *< 0.005 and >2 SD fold-change expression) by chance is 0.005 × 5,031, or 25 probes. The actual number of probes meeting the combined threshold was 3,912, corresponding to an apparent FDR of 25/3,912, or 0.64%. Commonly used multiple testing correction methods such as Bonferroni or Holm step-down were not applied as these eliminate a large number of true positives and introduce an inappropriate over correction.

A system of binary and decimal flags was used to cluster the differentially regulated genes into subgroups based on expression ratios [18]. Briefly, all genes that met both the fold change and the statistical significance threshold criteria (average ratio >2, and *p *< 0.005) for one or more of the three 5 mM MAA treatment conditions were assigned a binary flag value of 1, 2, 4 respectively. The sum of these binary flag values defines the whole number portion of the flag assigned to each gene and indicates which of the three microarrays met the specified threshold criteria in our analysis. In addition, decimal values of 0.1, 0.01, 0.001 or 0.2, 0.02, 0.002, were respectively assigned to each of the three microarrays to indicate the direction of regulation of the genes in the array (decimal flags with values of 1 indicate up-regulation, whereas those with a value of 2 indicate down-regulation). Thus, for each gene, the Total Flag Sum (TFS), comprising the binary sum plus the decimal values, indicates which of the three arrays met the threshold criteria for inclusion and the direction of regulation. A similar flag system was used to identify common response genes at 1 mM and 5 mM, with by extending the TFS to six binary flags (1, 2, 4, 8, 16 and 32) and six decimal values (0.1, 0.01, 0.001, 0.0001, 0.00001, 0.000001 or 0.2, 0.02, 0.002, 0.0002,0.00002, 0.000002), except that in that case the average ratio threshold was set at >2SD, corresponding to a fold change of >2-fold for the 5 mM MAA data set and >1.5-fold for the 1 mM MAA data set.

Principal component analysis was used to extract characteristic patterns from the six microarray data sets. 5624 genes responding to either 1 mM MAA or 5 mM MAA were selected based on the combined criteria of |fold-change| >2 SD from mean and *p *< 0.005 at one or more time points. The data were then pre-processed by logarithm 2 transformation of the expression ratio for each gene and by normalizing each gene's ratio to a mean value of zero and to a SD of 1 across the set of 6 arrays. Matrix *A*, which represents the gene expression data under all 6 microarray conditions, was decomposed by the singular value decomposition: *A *= *U∑V^T^*, where both *U *(an *N *× *M *matrix) and *V *(an *M *× *M *matrix) are orthogonal matrices, and *∑ *(an *M *× *M *matrix) is diagonal. The loading matrix *V *consists of the weights of individual genes in the principal components. V^*T*^ is the transpose of V. We denote *X *= *U∑*, where the principal component scores are specified.

### Motif enrichment analysis

Flexmodule_motif, a *de novo *motif discovery function based on Gibbs motif sampler implemented in CisGenome [19], was used to identify DNA motifs overrepresented within 1 kb upstream of the transcription start sites of sets of genes either up or down regulated by MAA. The DNA motifs that best matched those overrepresented motifs were identified using STAMP, a database for alignment and similarity search for DNA motifs [20]. Next, Fisher's exact test was applied to test whether these known motifs are enriched in genes of each group compared to control gene sets. Two sets of genes were used as background controls: 1) genes expressed at a modest or high level in untreated TM3 cells (microarray signal intensity > 200) that were not responsive to MAA treatment (|expression fold-change| < 1.2 following 5 mM MAA treatment at 3, 8 and 24 h); and 2) genes expressed in TM3 cells at a low to undetectable level (microarray signal intensity <50) and |expression fold-change| <1.2 at all three time points following MAA treatment.

### Ingenuity Pathway Analysis, GO enrichment and heat maps

The list of MAA-regulated genes was analyzed using Ingenuity Pathway Analysis software (Ingenuity Systems, Mountain View, CA) based on gene ontology, biological processes, molecular function, and genetic networks. This software maps the biological relationship of uploaded genes into networks based on published literature for each gene. The biological function network, for example, identifies biological functions and diseases that are most significant to the data set. Genes that met the threshold criteria for MAA responsiveness and were associated with biological functions or diseases in the Ingenuity Pathway knowledge base were considered for further analysis. Fischer's exact test was used to calculate a *p*-value to determine the probability that each biological function/disease assigned to the data set is due to chance alone. A relevancy score was assigned to each network in the data set to estimate the relevancy of the network to the gene list uploaded. A higher relevancy score means that the network is more relevant to the gene list entered. Top pathways in each network are listed according to their *p*-values. Classified gene lists were analyzed for enrichment of Gene Ontology functional annotation clusters using DAVID, a web accessible bioinformatics database [21,22]. Heat maps were drawn using Java Treeview [23].

## Results

### Overall impact of MAA on TM3 cell gene expression

The impact of MAA treatment on mouse TM3 cell gene expression was determined in cells treated with either 1 mM or 5 mM MAA for 3, 8 or 24 h. These concentrations of MAA were chosen based on earlier reports demonstrating similar MAA concentrations in plasma of rodents exposed to toxic doses of ethylene glycol monomethyl ether [10] and in urine of humans exposed to ethylene glycol monomethyl ether in occupational situations [11]. Under these conditions, MAA did not cause any loss of TM3 cell viability over the course of at least 48 hr. The three time points were selected to distinguish early MAA response genes (putative direct targets, whose expression does not require *de novo *protein synthesis) from late response genes (secondary response genes) [24]. RNA samples representing each time point were analyzed on expression microarrays in direct comparison to vehicle-treated controls.

**Figure 1:**
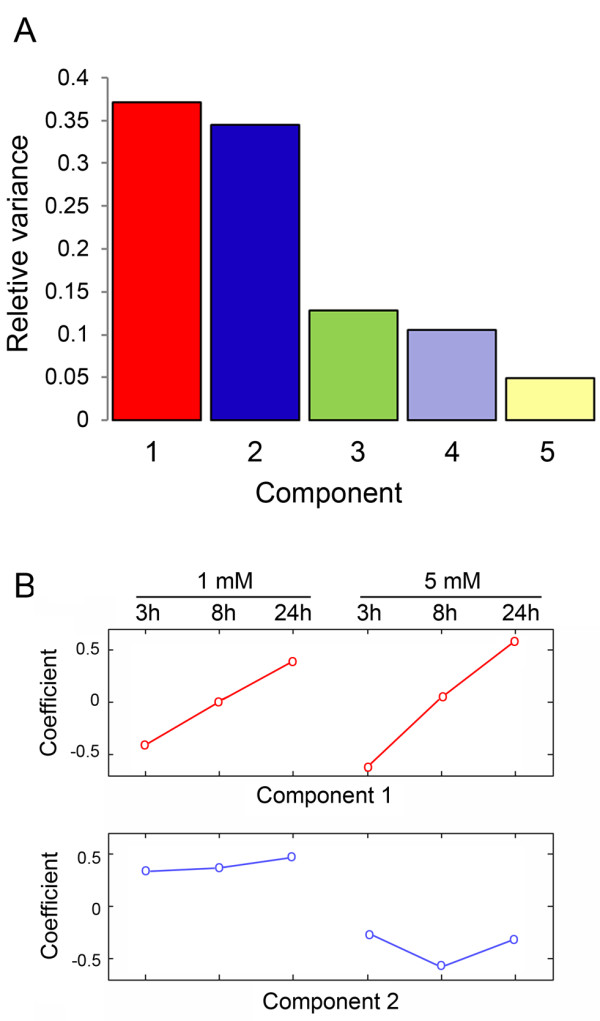
**Principal component analysis of six microarray data sets**. Plots of relative variance (A) and the coefficients of first and second components (B) are shown. Each coefficient indicates the weight of a particular treatment in the principal component. The first principal component represents a measure of expression change over time. The second coefficient captures information about the expression pattern affected by MAA dosage.

**Figure 2:**
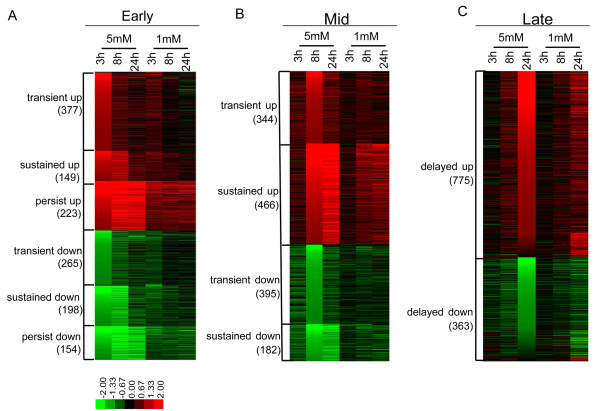
**Heat map for MAA response genes**. Shown are the expression profiles of early (A), mid (B) and late (C) MAA response genes based on log 2 ratios. The numbers of transcripts in each subgroup is marked in parenthesis.

Principal component analysis was applied to evaluate the expression patterns in the six microarrays. The first two of 6 principal components account for more than 70% of the variance in the overall data set (Fig. 1A). The pattern of the first component represents genes showing a steady increase in expression, or a steady decrease in expression, at both MAA concentrations (Fig. 1B), while the second component represents genes that show small changes in expression as a function of time at 1 mM MAA as compared to 5 mM MAA (Fig. 1B and Additional file 1). Based on these analyses, 37% of the variance in the data can be explained by the treatment duration, while 34% of the variance is due to different responses at low vs. high MAA concentrations. Most of the genes that show a different response at each MAA concentration show a more significant response to 5 mM MAA than 1 mM MAA, as would be expected (Fig. 2 and Additional file 1). To explore the genes and pathways that may potentially contribute to MAA toxicity, we focused our analysis on the effect of 5 mM MAA, and describe below common pathways triggered by both 1 mM and 5 mM MAA.

3,912 of the 41,174 microarray probes met the threshold criteria (absolute log 2 ratio >2 SD above the mean and *p *< 0.005), corresponding to an apparent false discovery rate (FDR) of 0.64%, for at least one of the three 5 mM MAA time points after elimination of redundant probes. The levels of 1,382, 2,122 and 2,226 transcripts were significantly altered by MAA at 3 h, 8 h and 24 h, respectively. A complete list of the MAA-responsive genes together with their expression ratios and fluorescence signal intensities at each time point is provided in Additional file 2. Ingenuity Pathway Analysis was used to map the biological relationship of the MAA-responsive genes into networks constructed based on published literature about each gene (Table 1). Gene networks that responded to MAA include reproductive system development and function, embryonic development and tissue morphology. Disease categories that were impacted by MAA include reproductive system disease, inflammatory disease, inflammatory response, connective tissue disorders and skeletal and muscular disorders. The biological functions affected by MAA include cellular movement, cell-to-cell signaling and interaction, cellular development and cell death (Table 1).

**Table 1:** Top biological functions and networks affected by MAA

Name	*p*-value range	No. molecules
**A. Top Biological Functions: Diseases and Disorders**		
Reproductive System Disease	3.45E-10 - 4.68E-04	160
Inflammatory Disease	1.76E-09 - 1.23E-03	507
Inflammatory Response	1.18E-08 - 1.77E-03	279
Connective Tissue Disorders	5.10E-07 - 1.23E-03	44
Skeletal and Muscular Disorders	5.10E-07 - 1.23E-03	516
**B. Molecular and Cellular Functions**		
Cellular Movement	7.17E-12 - 1.81E-03	327
Cell-To-Cell Signaling and Interaction	3.03E-11 - 1.53E-03	333
Cellular Growth and Proliferation	3.74E-10 - 1.75E-03	492
Cellular Development	7.76E-08 - 1.81E-03	455
Cell Death	8.78E-07 - 1.69E-03	490
**C. Physiological System Development and Function**		
Tissue Development	3.47E-10 - 1.53E-03	353
Cardiovascular System Development & Function	5.55E-08 - 1.56E-03	169
Organismal Development	5.55E-08 - 1.36E-03	240
Hematological System Development & Function	7.98E-08 - 1.75E-03	348
Immune Cell Trafficking	1.58E-07 - 1.77E-03	206
**D. Associated Network Functions ***(top five gene network functions)*		
1. Reproductive System Development and Function, Embryonic Development, Tissue Morphology.		
2. Infection Mechanism, Dermatological Diseases and Conditions, Organismal Injury and Abnormalities.		
3. Carbohydrate Metabolism, Small Molecule Biochemistry, Molecular Transport.		
4. Behavior, Nervous System Development and Function, Gene Expression.		
5. Cardiovascular System Development and Function, Embryonic Development, Tissue Development.		

Shown is a summary of results of Ingenuity Pathway bio-functional analysis to identify biological functions or disease networks that are most significant to the 5 mM MAA data set. Genes that met the threshold criteria defined in Materials and Methods and were associated with biological functions or diseases in the Ingenuity Pathway Analysis Knowledge Base were considered in the analysis. Each network includes a set of molecules, which usually perform more than one function. Fischer's exact test was used to calculate a *p*-value indicating the probability that each biological function and/or disease assigned to the data set is due to chance alone. Shown is the *p*-value range, which indicates the lowest and highest *p*-values corresponding to all molecules included in each network, and the total number of molecules present in the network.

### Clustering and functional analysis by significance and differential expression

**Figure 3:**
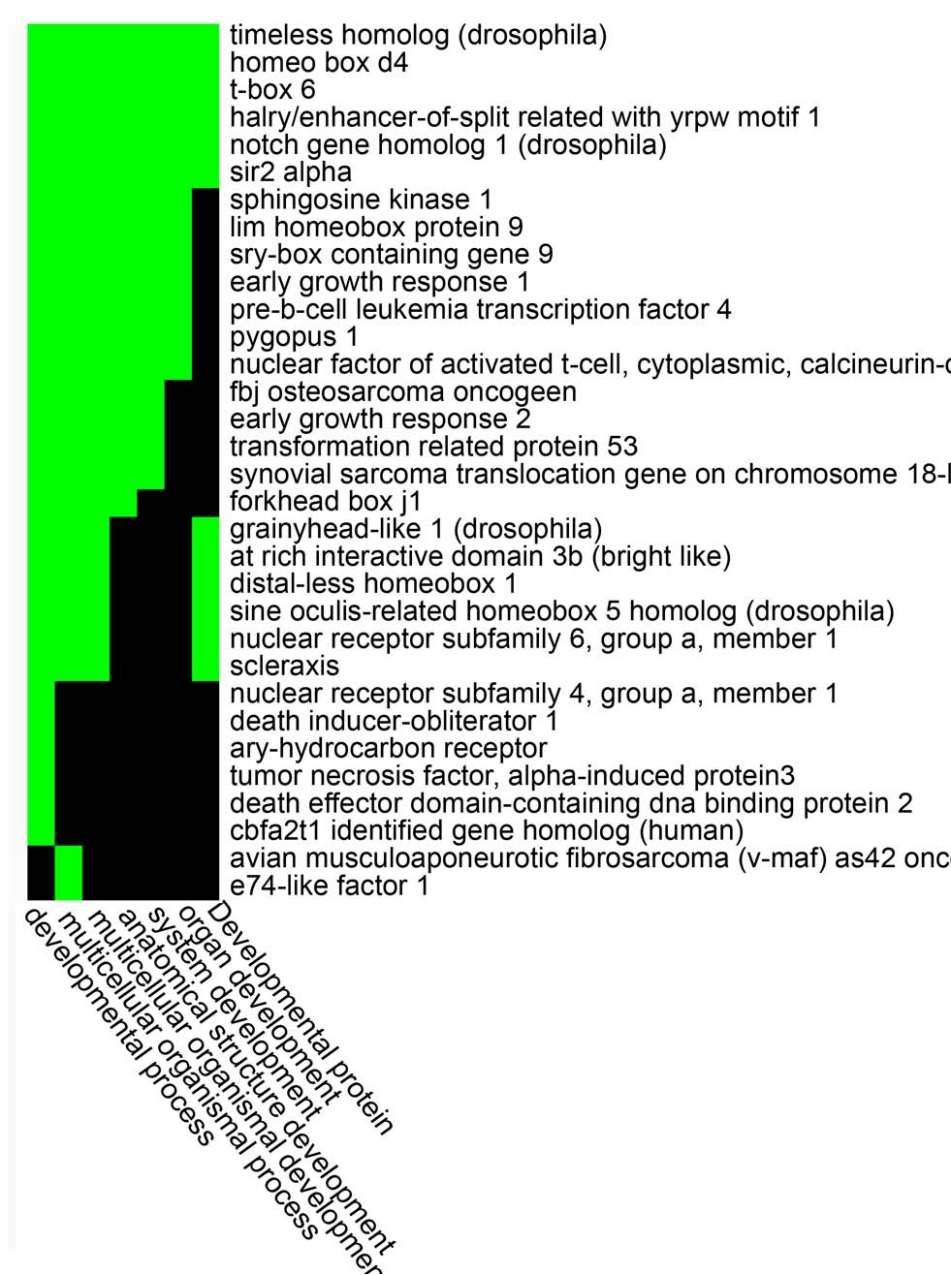
**32 DNA-binding early MAA response genes that are associated with developmental processes**. Each column represents a GO term associated with development process; genes (indicated by rows) with specific GO terms are marked with green.

The impact of MAA treatment was further investigated by classification of the 3,912 MAA-responsive genes using a binary flagging system termed Total Flag Sum (TFS) [18], whereby each gene is categorized based upon its expression ratio and *p*-value in each of the three microarray experiments. Genes that responded to MAA rapidly, i.e., by the 3 h MAA time point, were distinguished from the mid-response genes (response by 8 h) and the late responders (response by 24 h) (Table 2 and Additional file 2). A total of 1,366 early response genes were identified, which were further categorized based on the persistence of their response to MAA (Table 2 and Fig. 2A). Among the early response genes, 642 genes responded at the 3 h time point but not at 8 h (early transient response genes), with 377 transcripts induced and 265 transcripts repressed. 106 of these early transient genes participate in transcriptional regulation, as determined by gene functional annotation analysis using DAVID [21,22] (Table 3 and Additional file 3), and could contribute to the complex changes in the transcriptome associated with MAA treatment. Indeed, 102 of the 1,366 early response genes encode DNA-binding proteins, 60 of which showed a transient response to MAA (Additional file 4). Interestingly, 32 of these 102 early response DNA-binding proteins have been linked to developmental processes by DAVID analysis (Fig. 3). Moreover, 31 of the 102 genes contain zinc finger domains (Additional file 4). Functions have not been determined for many of these genes; however, two of the induced zinc-finger protein genes, *Egr1 *and *Egr2*, are notable insofar as *Egr1-Egr4 *double-mutant male mice are characterized by physiologically low serum testosterone levels and atrophy of androgen-dependent organs due to the loss of luteinizing hormone production in the pituitary gland [25]. Moreover, Egr1 is a key transcription factor implicated in the development and progression of prostate cancer [26].

**Table 2:** Classification of MAA responsive genes by duration of MAA treatment required to elicit response

Group	Sub-group	3 h MAA	8 h MAA	24 h MAA	Genes in each subgroup (number)
**Early (1366 genes)**	early transient up	Up	-	-	351
	early transient up	Up	-	Up	26
	early sustained up	Up	Up	-	149
	early persistent up	Up	Up	Up	223
	early transient down	Down	-	-	245
	early transient down	Down	-	Down	20
	early sustained down	Down	Down	-	198
	early persistent down	Down	Down	Down	154
**Mid (1387 genes)**	mid transient up	-	Up	-	344
	mid sustained up	-	Up	Up	466
	mid transient down	-	Down	-	395
	mid sustained down	-	Down	Down	182
**Late (1138 genes)**	late up	-	-	Up	775
	late down	-	-	Down	363

The 5 mM MAA-responsive genes were divided into 3 groups, early response (3 h), mid response (8 h) and late response (24 h) based on the minimum period of MAA exposure that was required to evoke a gene response compared to control cells. The direction of response is indicated as Up (up-regulation), Down (down-regulation) or - (no significant response). 21 other genes that did not meet these MAA response criteria are shown in Additional file 2.

**Table 3:** Gene functional clusters affected by MAA for each sub-group

Response	Top term	Enrich-ment score	Cluster gene count	*p*-value
**Early transient**	GO:0045449, Regulation of transcription	5.96	106	3.23E-07
	GO:0044424, Intracellular part	5.8	211	1.11E-06
	GO:0065007, Biological regulation	5.54	124	6.15E-07
				
**Early sustained**	GO:0050794, Regulation of cellular process	3.41	66	1.85E-04
	IPR000867: Insulin-like growth factor-binding protein, IGFBP	3.04	4	6.28E-04
	Domain: von Willebrand factor, type C	2.91	4	4.81E-04
				
**Early persistent**	GO:0048731, System development	2.19	48	1.19E-03
	GO:0048514, Blood vessel morphogenesis	2.13	9	2.17E-03
	GO:0044421, Extracellular region part	2.07	43	6.46E-03
				
**Mid transient**	GO:0007155, Cell adhesion	3.11	34	3.28E-04
	GO:0044237, Cellular metabolic process	2.25	347	1.60E-04
	IPR004021: HIN-200/IF120x	2.13	5	9.10E-04
				
**Mid sustained**	GO:0044421, Extracellular region part	8.22	109	7.30E-10
	IPR015493: Protocadherin beta	6.56	15	1.68E-12
	IPR003128: Villin headpiece	4.39	5	2.85E-05
				
**Late**	GO:0044421, Extracellular region part	10.34	165	5.60E-12
	GO:0048856, Anatomical structure development	4.4	144	1.10E-05
	GO:0044449, Contractile fiber part	3.47	12	1.75E-04

Listed are the top three gene functional clusters affected by MAA as determined using DAVID database. The top term for each cluster, the cluster enrichment score and the number of regulated genes in each cluster are listed.

Early response genes whose expression was altered by MAA at 3 h and 8 h, but not at 24 h, were designated early sustained up or early sustained down genes (Table 2 and Additional file 2; TFS groups 3.110 and 3.220, respectively). These genes are enriched in several cancer-related functional clusters, including insulin-like growth factor binding (*Htra3, Igfbp5, Cyr61, Wisp1*), GTP binding (*Tubb6, Tgtp, Rnd1, Gbp1, Rab40b, Gbp2, Rhobtb1, Rabl3, Gbp3*), cytokine activity (*Clcf1, Kitl, Socs2, Cxcl5, Tnfsf11, Cxcl1, Cx3cl1, Il12a*) and negative regulation of apoptosis (*Cln8, Clcf1, Nuak2, Kitl, Dlx1, Notch1, Bcl2, Serpinb9, Clec2 d, Nr4a1, Fastkd2, Rassf5, Elmo1*) (see Additional file 3 for complete listing of genes). Corresponding KEGG pathways include cytokine and receptor interaction, TLR-Jak-STAT signaling pathway, and phosphatidylinositol signaling. Other early genes whose response to MAA was maintained at 8 h and 24 h were designated early persistent genes (Table 3 and Additional file 2; TFS groups 3.111 and 3.222); examples include genes implicated in cytokine receptor interaction and calcium signaling (Additional files 3 and 5).

1,387 mid-response genes responded to MAA at 8 h, while 1,138 late-response genes did not respond to MAA until 24 h (Figs. 2B, C and Table 2). These secondary response genes are enriched in a variety of signaling pathways and functional clusters, such as macromolecule metabolic process (*Atf3, Adra1b, Fbxl16, Frk, Gls, Nanos1, Pcyt1b*), cell adhesion (*Arhgap6, Col1a2, Col6a2, Kitl, Tek, Thbs1, Tsc1*), interferon-activated genes (*Ifi202b, Ifi203, Ifi204, Ifi205*), genes active in the extracellular space (*Acp5, Csf1r, Muc2, Nppb, Ramp3*), T cell activation and differentiation (*Cd74, H2-DMa, Il15, Il7, Itgal*) and metabolism of xenobiotics by cytochrome P450 and other enzymes (see Additional file 3 for complete listing of genes).

### DNA motifs associated with TM3 cell transcriptional responses

CisGenome [19] was used to discover *de novo *motifs overrepresented in the sets of genes up or down regulated genes by MAA at each time point. GC rich motifs were found to be over represented in the proximal promoters of MAA up regulated genes. Motif comparison against the TRANSFAC database [27] revealed that these GC rich motifs are most similar to two known motifs, SP1 and KROX, which correspond to the binding sites for transcription factors of the SP1 and EGR families, respectively. Both motifs are enriched in MAA-regulated genes compared to a background gene set comprised of TM3 cell non-expressed genes, with enrichment scores ranging up to 2.1 (Additional file 6). However, motif enrichment was not observed when using as background a set of TM3 cell expressed genes that did not respond to MAA (Additional file 6). Thus, while SP1 and KROX/EGR motifs are overrepresented in some MAA-induced genes, these motifs also characterize TM3 cell expressed genes more generally.

### Impact of MAA on genes associated with testicular function

**Figure 4:**
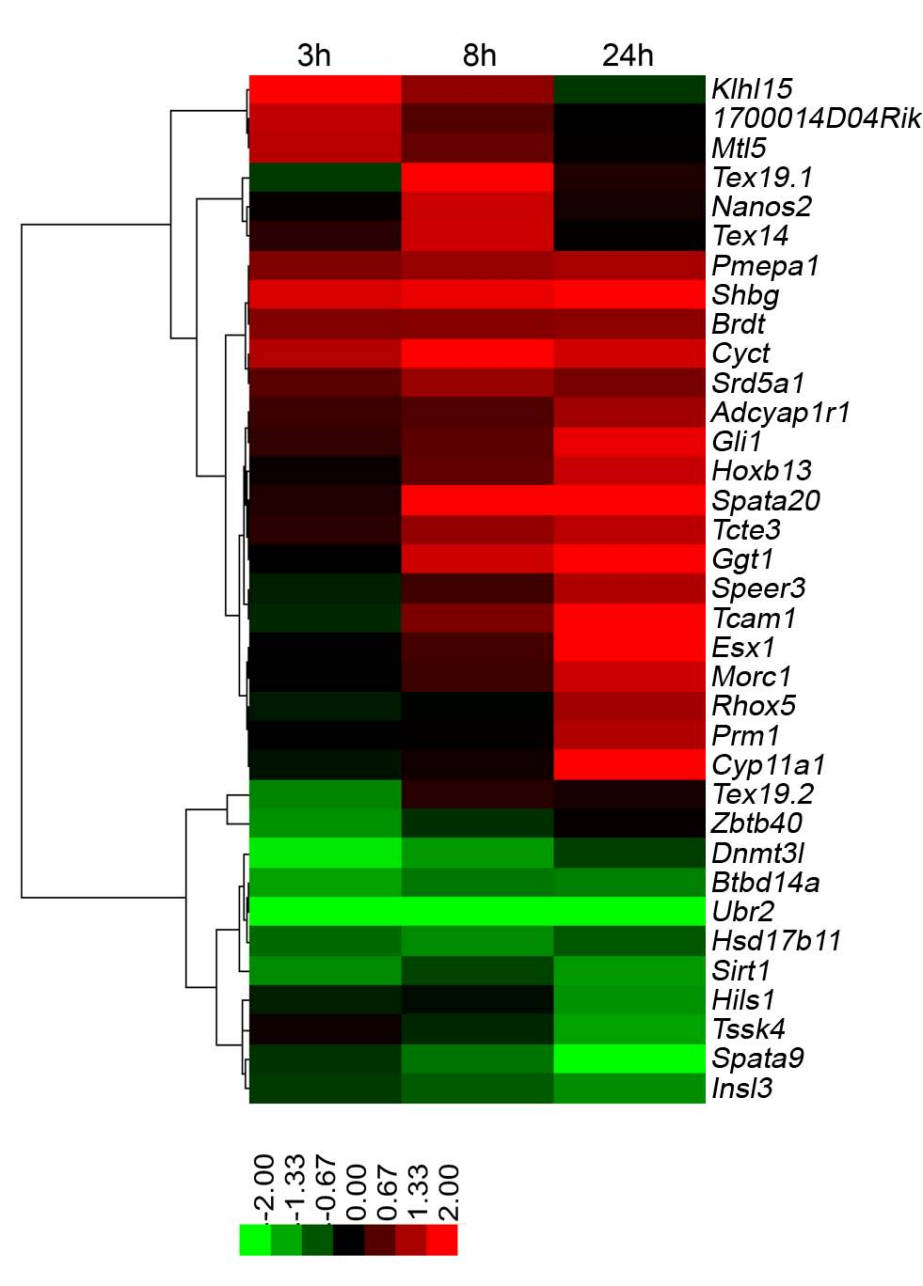
**Heat map presenting the effects of MAA treatment for 3, 8 and 24 hr on genes related to testicular function**. Shown is the hierarchical clustering heat map for 35 testis-associated genes based on log 2 ratios (scale is as shown at the bottom).

MAA had a wide range of effects on gene products important for testosterone biosynthesis, spermatogenesis, and testicular development and function (Fig. 4 and Additional file 7). For example, sex hormone binding globulin (*Shbg*) was up regulated by 5 mM MAA at all 3 time points, while the induction of *Cyp11a1, Rhox5 (Pem) *and *Hoxb13 *and the repression of *Insl3 *were seen at the 24 h MAA time point.

### Common response genes and pathways activated at low MAA concentration

Early, mid and late MAA response genes were also identified in the 1 mM MAA-treated TM3 cells. Early response genes common to the 1 mM and 5 mM MAA treatments (482 genes; Additional file 8) are expected to be enriched in direct targets of MAA; 294 of these genes were induced and 188 were repressed by MAA. DAVID analysis showed that phosphatidylinositol/phospholipase C/calcium signaling and cytokine/cytokine receptor interactions were the most significantly enriched pathways for the common early gene set. Pathways enriched in the common mid and late response genes include cell adhesion and focal adhesion (Additional file 9).

### Real time qPCR validation

**Figure 5:**
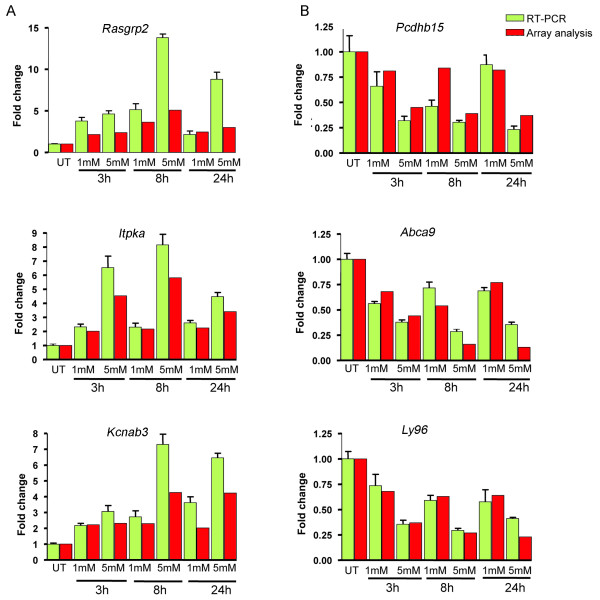
**qPCR analysis of select genes**. Shown are qPCR data for three genes induced at all three time points by both 1 mM and 5 mM MAA (panel A; *Rasgrp2*, *Itpka *and *Kcnab328*) and three genes repressed by 5 mM MAA at all three time points (panel B; *Pcdhb15*, *Abca9 *and *Ly96*). qPCR primers used for this analysis are shown in Additional file 10.

To confirm the results of the microarrays, qPCR analysis was performed for three genes that were induced (*Rasgrp2*, *Itpka *and *Kcnab328; *Fig. 5A) and for three genes that were repressed by MAA at all three time points (*Pcdhb15*, *Abca9 *and *Ly96*; Fig. 5B). *Rasgrp2 *and *Itpka *were of particular interest since our earlier studies had shown that MAA induces the Map kinase pathway and the PI3 kinase pathway in tsA201 cells [9]. Fig. 5 shows that the overall trends of expression seen by qPCR were consistent with the microarray data, although the fold-changes did not always match those of the microarray data, as is commonly seen.

## Discussion

MAA is the active metabolite of the industrial chemical ethylene glycol monomethyl ether, a widely studied testicular toxicant. Presently, we characterize changes in gene expression induced by MAA in the cultured testicular Leydig cell model TM3. This investigation, carried out as a time course of MAA exposure, was designed to gain further insight into the range of changes in gene expression that MAA induces, including gene responses that could contribute to the testicular toxicity that is a hallmark of MAA exposure. The TM3 cell line was chosen based on our earlier finding that these cells are responsive to MAA, which induces changes in the expression of several genes related to androgen synthesis and activity [9]. MAA did not cause any changes in TM3 cell viability over the course of at least 48 hr; nevertheless, we observed extensive changes in TM3 cell gene expression. 3,912 genes were altered in their expression by 5 mM MAA, the plasma MAA concentration associated with germ cell toxicity in mice [10]; 1,168 of these genes responded in common to 1 mM MAA, which is more relevant to the exposure level seen in humans [11]. As discussed below, the gene expression changes and associated cellular pathways impacted by MAA in this cellular model parallel some of the toxicities associated with MAA exposure, suggesting that these findings in TM3 cells may serve as a model for MAA-induced toxicities in other cell types as well.

### Rapid MAA induction of zinc finger transcription factors

Analysis of the time dependence of MAA-induced gene responses allowed us to identify genes that respond to MAA rapidly (within 3 h), as well as genes whose altered in expression persists and could potentially serve as biomarkers of MAA exposure. Environmental chemical-induced toxicities are often associated with early gene responses [28]; therefore, genes that respond to MAA rapidly may provide insights into the pathophysiological changes caused by MAA. Presently, we found that 102 of the 1,366 early response genes encode DNA-binding proteins, 32 of which have been linked to developmental processes (Fig. 3). Interestingly, 60 of the early response DNA-binding proteins showed a transient response to MAA, i.e., gene induction responses seen at 3 h were largely reversed by 8 h (Additional file 4), suggesting a feedback loop for the regulation of transcriptional activity by MAA. For example, ATF1 (activating transcription factor 1), a bZIP domain containing protein belonging to the CREB family, was down regulated 2-fold after 3 h of MAA treatment, while CREB5 (cAMP response element-binding 5), another CREB family transcription factor, was up regulated 3.7-fold. Upon activation, CREB binds as a dimer to the cAMP response element (CRE), where it promotes the recruitment of the transcriptional coactivators CREB binding protein (CBP) and p300 [29]. The impact of this opposite regulation of these two CREB family proteins by MAA on the expression of CRE-regulated genes is unknown. One CRE-regulated gene, *Egr1*, was induced 4.5-fold after 3 h MAA exposure. EGR1 is a zinc-finger transcription factor that binds to a GC-rich motif, as do several other zinc finger proteins, which may activate or repress their targets by acting in combination [30]. Previously, evolutionarily conserved zinc finger transcription factor binding sites recognized by WT1, EGR1, SP1, SP2, AP2 and GATA1 were identified in the promoters of 24 differentially expressed prostate cancer genes from eight mammalian species [26], suggesting these zinc finger proteins play a pivotal role in the prostate. Here, both EGR1 and SP1 binding sites were found to be overrepresented in TM3 Leydig cell-expressed genes compared to TM3 cell non-expressed genes. Further studies, including gene knock-down and *in vivo *transcription factor binding assays, are needed to clarify whether these putative sites are true EGR1/SP1 binding sites, whether they are functional, and what impact their binding may have on TM3 cell gene activation or repression.

### Impact of MAA on protein kinase pathways

Protein kinases play a significant role in MAA-induced toxicity, and protein kinase inhibitors can minimize these effects [31]. Of the 3,912 MAA-regulated genes identified in our study, 91 code for protein kinases, as determined by Ingenuity Pathway Analysis (Additional file 11). These kinases are associated with amino acid metabolism, post-translational modification, small molecule biochemistry, cell death and cell movement. The top canonical pathways affected include inositol metabolism, axonal guidance signaling, FAK signaling, germ cell-Sertoli cell junction signaling, and nicotinate and nicotinamide metabolism. MAA can act as a hormone sensitizer to enhance the transcriptional activity of several nuclear receptors without itself being a hormone mimetic, although the underlying mechanism is only partly understood [9,32]. Our previous studies demonstrated that the potentiation of androgen receptor activity by MAA requires tyrosine kinase signaling that is independent of the RAS-MEK-ERK signaling pathway but requires PI3 kinase activity [9]. Consistently, in this study, we observed that the phosphoinositide/phospholipase C/calcium pathway is enriched in the set of early response genes in common to both 1 mM and 5 mM MAA treatment. Several key factors in this pathway are induced by MAA, including phospholipases C, PI3 kinases, and IP3 receptor (Additional file 9). Both the ERK and PI3 kinase pathways have been suggested to be responsible for *Egr1 *up regulation [33,34]. Further studies will be required to identify the underlying molecular mechanism whereby MAA activates these signaling pathways leading to induction of *Egr1 *and its downstream targets.

### Effect of MAA treatment on histone genes

Several genes encoding histone proteins were induced by MAA treatment. Thus, *Hist1h1d, Hist1h4k*, and *Hist2h2aa1 *were early MAA response genes, *Hist1h1c, Hist2h2be*, and *Hist3h2ba *were mid-response genes, and 9 other *Hist *genes, including 6 encoding histone 1 proteins, were late response genes (Additional file 2). H1 histones stabilize compact, higher order structures of chromatin, regulate gene expression, and participate in chromatin-based processes like DNA replication and repair [35]. The altered expression of this linker histone in MAA-treated cells could destabilize chromatin architecture and contribute to malignant transformation or genetic disorders. *Hist1h1d *is significantly up regulated in rat testis within 4 h of MAA treatment, and this change has been correlated with increased acetylation of core histones [36]. The induction of mouse *Hist1h1d *seen in our study is consistent with that earlier report, and supports the utility of the mouse TM3 model for identifying gene changes that may be linked to MAA toxicity. It will be interesting to determine the relationship between histone up regulation and the other gene expression changes caused by MAA.

### Biological pathways affected by MAA

Many of the prominent toxicological effects of MAA or the parent toxicant, ethylene glycol monomethyl ether, are well characterized [37] and several studies have elucidated effects of MAA on intracellular signaling pathways and changes in gene expression [3,36]. Using Ingenuity Pathway Analysis we identified pathways related to reproductive system development and function, embryonic development and tissue morphology to be the most affected by MAA. Moreover, at all three time points examined, MAA affected genes associated with several disease pathways, reproductive system disease, inflammatory disease, connective tissue disorders and skeletal and muscular disorders. Alteration of these pathways in the corresponding target tissues could contribute to some of the physiological diseases and disorders that have been associated with MAA exposure, including MAA-induced disruption of neurogenesis and limb and digit differentiation in mice [1], hematopoiesis [38], cell death [39] and immune system disorders [40-42]. MAA also induced significant changes in the expression of membrane proteins, which is reflected in the dysregulation of genes involved in cell-to-cell signaling, cell adhesion and cell mobility as indicated by Ingenuity Pathway functional analysis (Table 1). Indeed, a majority of the genes that were induced or repressed by MAA under all treatment conditions belong to this category (Table 3 and Additional file 3). Moreover, 32 of 102 early MAA response genes encoding DNA-binding proteins are associated with developmental processes (Fig. 3).

Genes related to testicular function whose expression was significantly altered by MAA include *Cyp11a1*, *Hsd17b11, Shbg *and *Insl3 *and *Rhox5 *(Fig. 4 and Additional file 7), which encode proteins involved in testosterone biosynthesis or development of the male reproductive system. Changes in the expression of these genes might impact testosterone biosynthesis/availability and influence spermatocyte survival. CYP11A1 cleaves the side chain of cholesterol to yield pregnenolone, the initial step in the pathway leading from cholesterol to steroid hormone production [43]. HSD17B11 is a hydroxysteroid dehydrogenase that is required for synthesis of androstenedione, a precursor of testosterone [44]. Insulin-like 3 (INSL3; Leydig insulin-like peptide) is important in testis descent [45], with male mice mutant for *Insl3 *exhibiting cryptorchidism or defects in testis descent due to abnormal gubernaculum development [46]. Steroid hormone binding globulin (SHBG) binds androgens and its over-expression can cause testosterone depletion and toxicity [47,48]. The expression of *Cyp17a1*, which catalyzes the 17-hydroxylase and lyase activities required for testosterone synthesis [49], was increased in MAA-treated cells, as determined by qPCR, although this change was not seen in the microarray analysis. Further studies are required to determine if these changes affect Leydig cell testosterone production, which could have a direct impact on germ cell survival and toxicity. MAA also induced two homeobox genes, *Rhox5 *and *Hoxb13*, which have been implicated in the modulation of androgen receptor-regulated gene expression in Sertoli cells and in prostate, respectively [50,51]. *Rhox5*, which is considered a Sertoli cell marker gene but is also expressed at a low level in Leydig cells *in vivo *[52], is thus an MAA-responsive transcription factor that may mediate some of the effects of MAA on downstream targets [50]. Finally, MAA down regulated estrogen receptor-α, suggesting the MAA can also impact estrogenic signaling in testicular cells. Of note, MAA also disrupts estrogenic signaling in MCF7 cells *in vitro *and in the mouse uterus *in vivo *[53].

Metabolic labeling studies establish that MAA can be activated to the thioester 2-methoxyacetyl- coenzyme A, which enters the tri-carboxylic acid cycle [1]. Conceivably, just as acetyl coenzyme A is funneled into multiple metabolic pathways, 2-methoxyacetyl-coenzyme A may enter multiple pathways, including tri-carboxylic acid cycle, fatty acid metabolism and amino acid metabolism with affects on cellular metabolism and gene expression. Acetyl coenzyme A is also essential for histone acetylation, a key event in gene transcription [54], suggesting that 2-methoxyacetyl-coenzyme A could also affect that process. We observed significant MAA-induced changes in expression of genes involved in cellular metabolism, oxidation status, transcription and gene expression, as might be caused by a metabolite which can enter and impact several key cellular metabolic and regulatory pathways. Further studies using inhibitors and metabolites of specific pathways are needed to test these hypotheses.

## Conclusions

In this study, we monitored the progressive changes in gene expression induced by MAA in a cultured Leydig cell model and detected extensive changes in TM3 cell gene expression. The MAA-responsive pathways identified are linked to reproductive system development and function, embryonic development and tissue morphology. These MAA-induced perturbations of cellular and biological functions may help elucidate the testicular pathophysiological responses induced by MAA exposure and identify useful biomarkers of MAA toxicity.

## Competing interests

The authors declare that they have no competing interests.

## Authors' contributions

GB and DJW conceived and designed the experiments, GB performed the experiments, YZ, GB and DJW analyzed the data and wrote the paper, and DJW managed the overall design and execution of the project. All authors read and approved the final manuscript.

## Supplementary Material

Additional file 1 **Principal component analysis of microarray data sets**. Click here for file

Additional file 2 **Expression ratio and TFS of 5 mM MAA response transcripts and transcripts of each subgroup**. Click here for file

Additional file 3 **Gene functional clusters affected by 5 mM MAA for each sub-group as determined using DAVID database, together with the KEGG pathways enriched in each group**. Click here for file

Additional file 4 **Expression ratio and TFS of 102 early response genes identified as DNA-binding proteins by their GO terms**. Click here for file

Additional file 5 **KEGG Pathways associated with 5 mM MAA early response genes**. In each pathway, the MAA-responsive genes are marked by a red asterisk. Click here for file

Additional file 6 **Enriched motif analysis of MAA response genes**. Click here for file

Additional file 7 **Impact of MAA on testis expressed genes**. Click here for file

Additional file 8 **Expression ratio and TFS of response transcripts common to both 1 mM and 5 mM MAA treatment in each sub-group**. Click here for file

Additional file 9 **Gene functional clusters and KEGG pathways enriched in each group of 1 mM and 5 mM MAA common response genes**. Click here for file

Additional file 10 **Mouse qPCR primer sets and Genebank accession numbers**. Click here for file

Additional file 11 **5 mM MAA regulated kinases as identified by Ingenuity Pathway Analysis (IPA)**. Click here for file
