# Supplementary material for: Impact of methoxyacetic acid on mouse Leydig cell gene expression
Source: Reprod Biol Endocrinol. 2010 Jun 18;8:65. doi: 10.1186/1477-7827-8-65 (PMC2909983; doi:10.1186/1477-7827-8-65)

Additional file 1. Principal component analysis of microarray data sets. Shown are the expression profiles of genes >97.5th percentile (or < 2.5th percentile) of the scores in each of the first two principal components shown in Fig. 1.

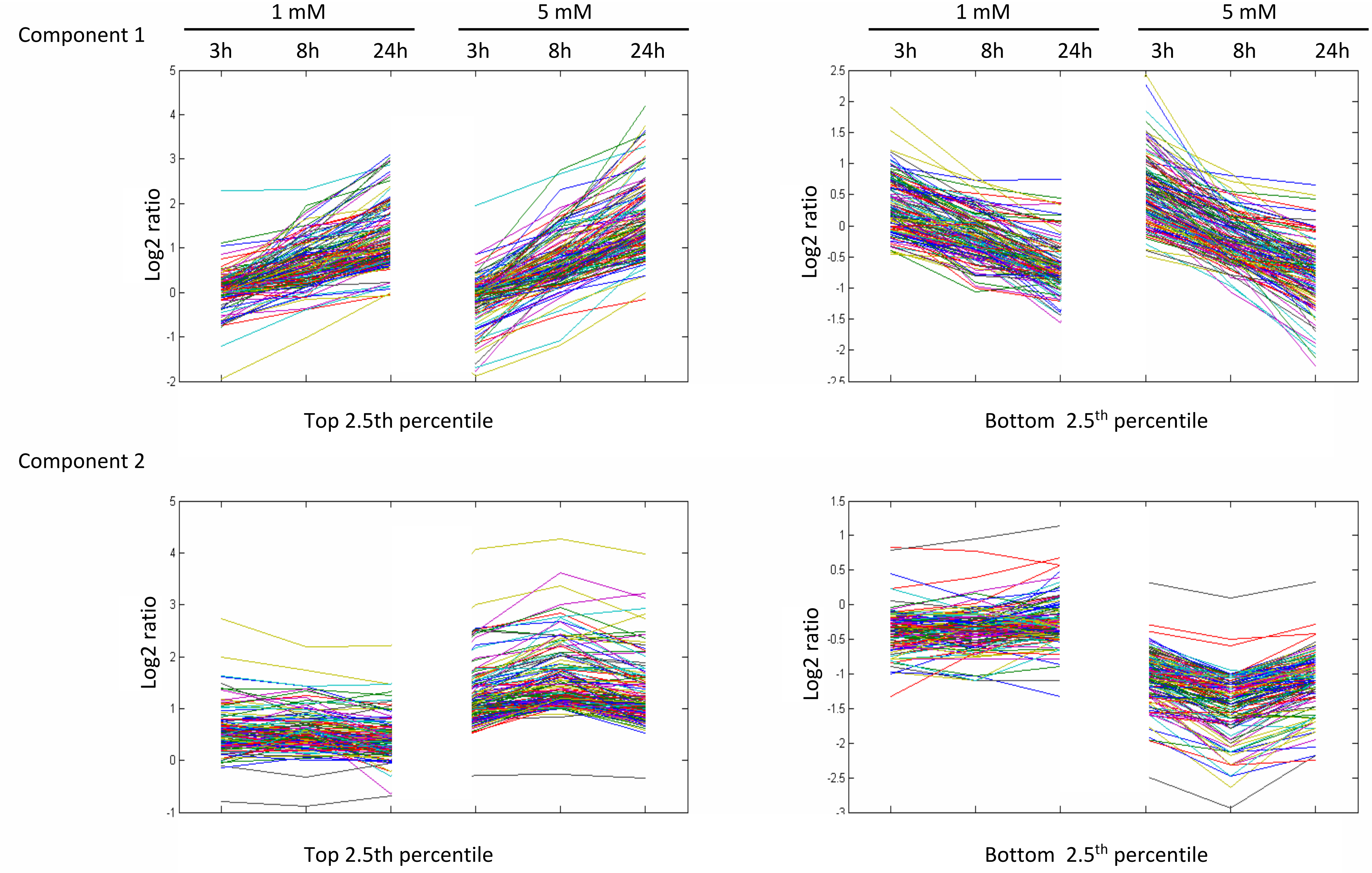

Supplement: Additional file 1 — Principal component analysis of microarray data sets. [file 1477-7827-8-65-S1.PDF]
