# Supplementary material for: Impact of methoxyacetic acid on mouse Leydig cell gene expression
Source: Reprod Biol Endocrinol. 2010 Jun 18;8:65. doi: 10.1186/1477-7827-8-65 (PMC2909983; doi:10.1186/1477-7827-8-65)

Additional file 5

KEGG Pathways associated with MAA (5 mM) early response genes

Cytokine-cytokine receptor interaction

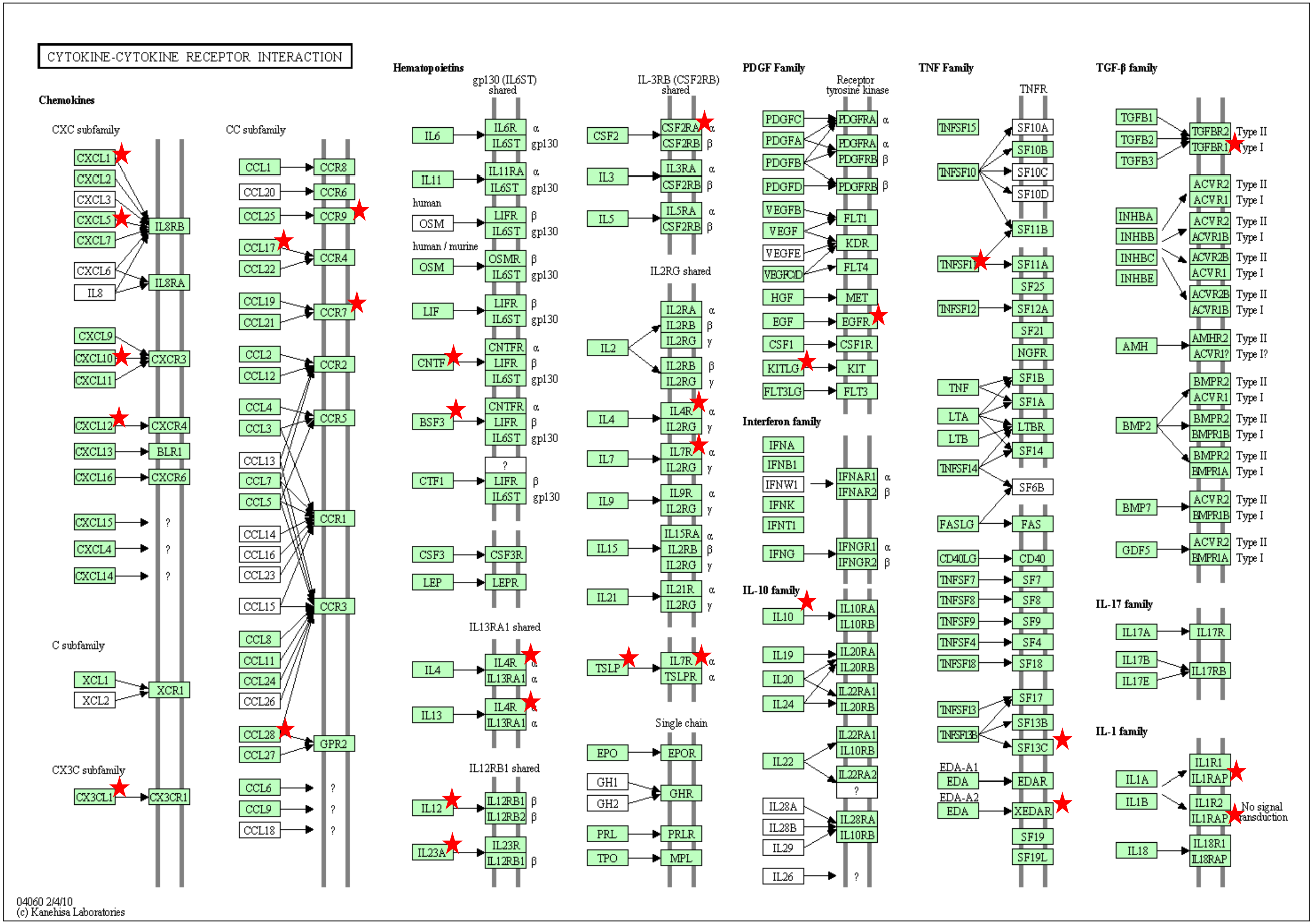

Calcium signaling pathway

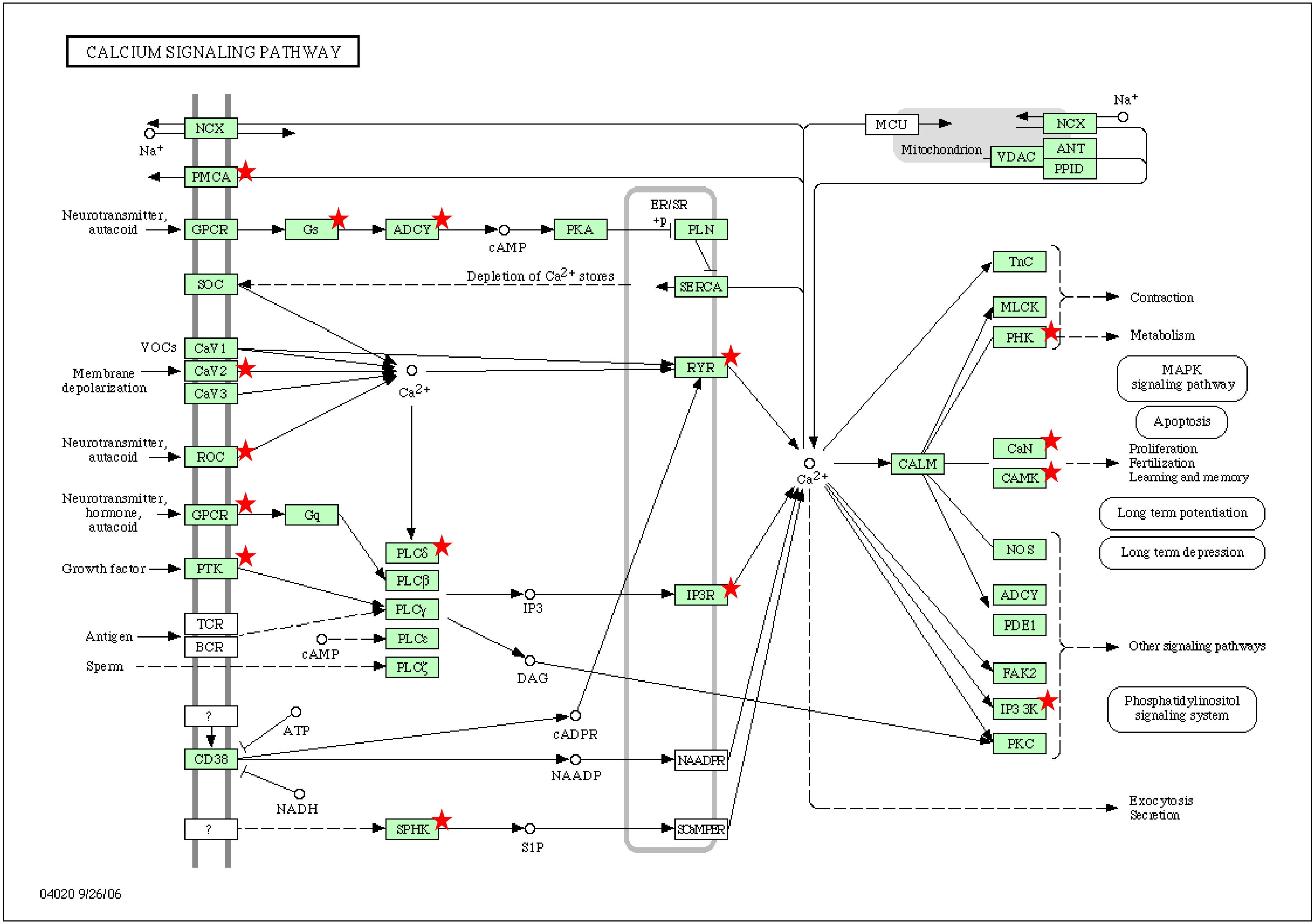

JAK-STAT signaling pathway

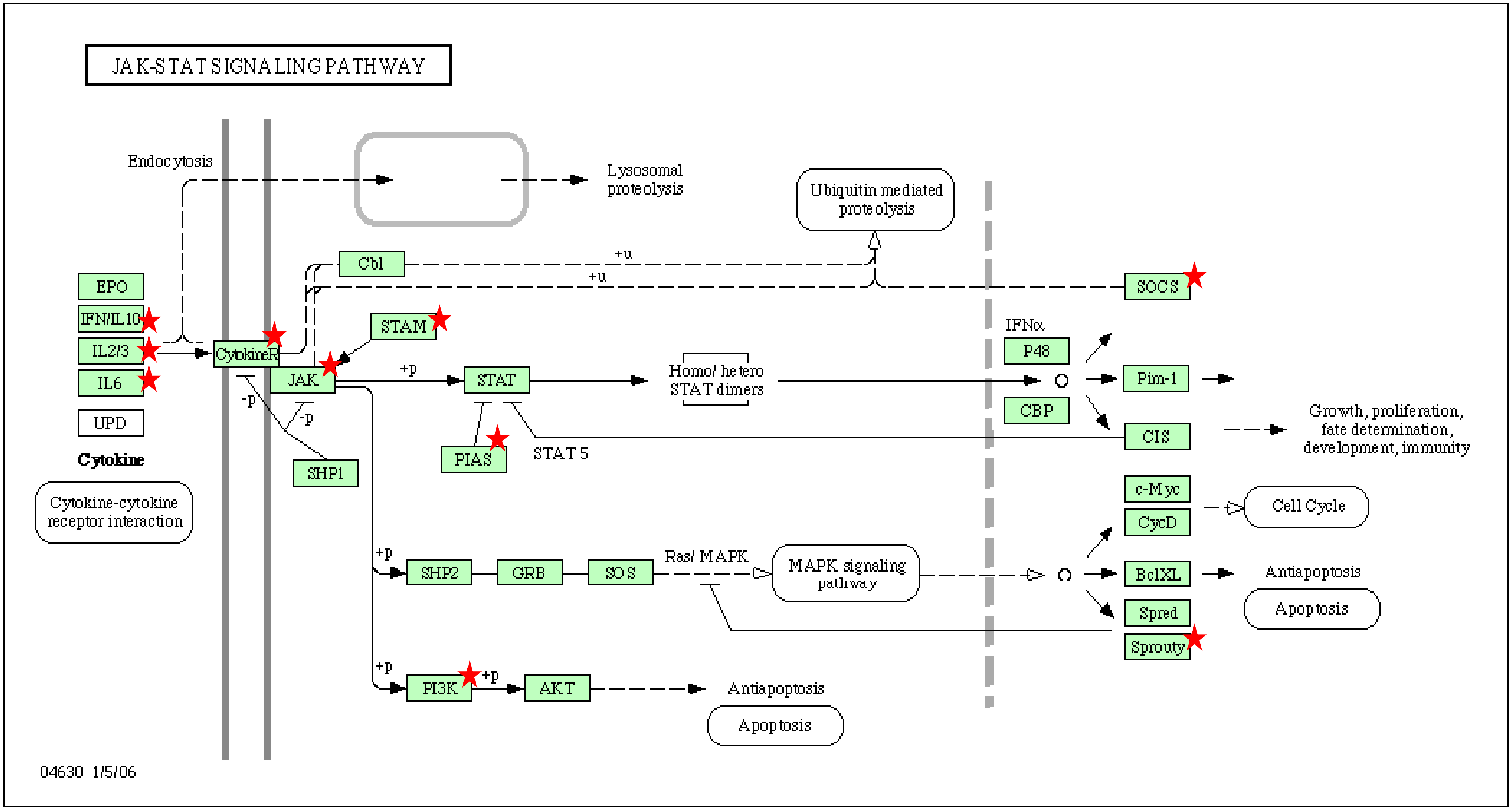

MAPK signaling pathway

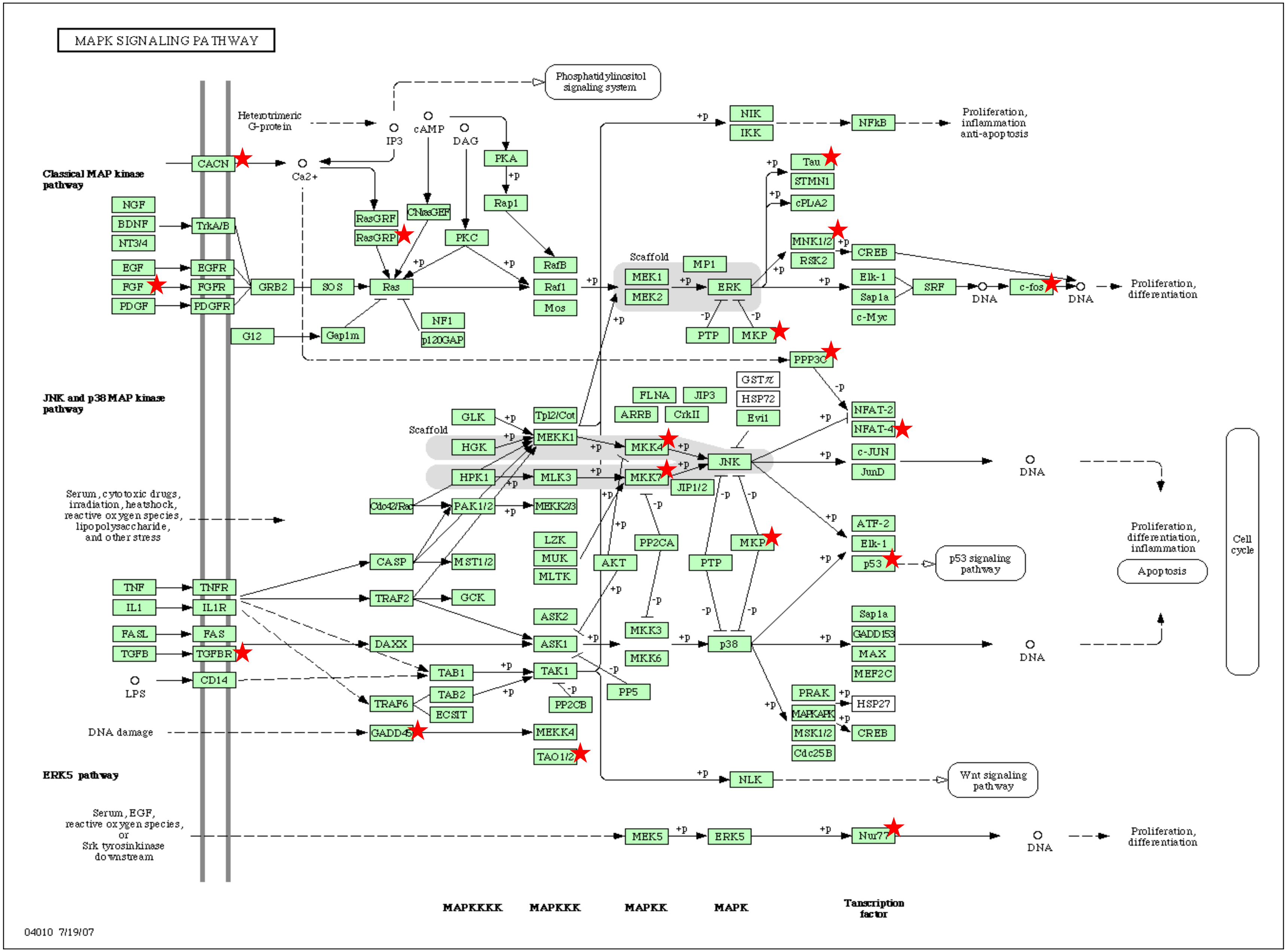

Supplement: Additional file 5 — KEGG Pathways associated with 5 mM MAA early response genes. In each pathway, the MAA-responsive genes are marked by a red asterisk. [file 1477-7827-8-65-S5.PDF]
